# Patients weigh in: The value of healthcare environmental stewardship to patient experience

**DOI:** 10.1016/j.joclim.2026.100647

**Published:** 2026-03-27

**Authors:** Shira R. Abeles, Jennifer Woods, Nicole Poletto, Lesley Wilson, Monica Nakielski

**Affiliations:** a University of California San Diego, USA; b University of California San Diego Health, USA

**Keywords:** Environmental stewardship, Patient experience

## Abstract

**Introduction:**

Human health relies on a healthy planet, yet the healthcare sector has a substantial negative impact on the environment, and ranks among the major greenhouse gas–emitting, plastic consuming, and waste generating sectors in the U.S. To better understand patient perspectives on this issue, University of California (UC) San Diego Health surveyed patients about the role of sustainability in healthcare.

**Methods:**

In March 2025, the UC San Diego Health Sustainability and Patient Experience teams conducted a brief patient opinion survey on the topic of environmental sustainability in healthcare. The survey was sent to patients who had previously volunteered to receive occasional surveys from UC San Diego Health.

**Results:**

Over 5,000 respondents (14.5 % response rate) reported overwhelming support for environmentally responsible practices in healthcare. The vast majority of respondents (94 %) viewed environmental health as either very or extremely important to their personal health, and 85 % agreed they would prefer to receive care from an organization committed to sustainability. Patients prioritized practices such as reducing waste and single-use plastics, transitioning to clean energy, and offering healthy, local food.

**Conclusion:**

These findings affirm that patients value alignment between healthcare delivery and environmental responsibility—an insight that offers additional motivation for healthcare systems to adopt and accelerate sustainable practices.

## 1 Introduction

In now-recognized irony, the healthcare industry is a major polluter and contributes significantly to climate change. This in turn exacerbates human illness and compromises the ability to provide healthcare due to increasingly destructive weather events disrupting access to care as well as supply chains for surgical, medical, and pharmaceutical supplies [[1], [2], [3], [4]]. Healthcare also consumes a significant amount of energy, water, plastics, single-use devices, pharmaceuticals, and other chemicals and materials, many of which unintentionally expose people to harm throughout their life cycles. The decline in human health due to increasing environmental harm is a significant concern to clinicians, the majority of voters in the U.S., and many nations globally [[5], [6], [7], [8], [9]]*.*

To align mission with operations, various health organizations and industries have initiated environmental stewardship programs to curb environmental harm and thus promote community health. Sustainable practices in healthcare include clean energy, energy efficiency, water conservation, waste reduction, sustainable purchasing, and minimizing the use of chemicals of concern.

Given the dire consequences predicted if human behavior does not change to address and mitigate climate change, and the highly visible resource consumption in U.S. healthcare, we sought to understand patient perspectives and priorities regarding environmental sustainability within healthcare [[10], [11], [12]]. Patient opinion and satisfaction is a critical component of assessing the value and direction of hospital operations, including sustainability programs. To that end, the University of California (UC) San Diego Health Sustainability and Patient Experience teams developed and distributed a survey to evaluate patients’ perceptions and preferences regarding environmental stewardship in healthcare.

## 2 Methods

The UC San Diego Health Patient Experience team regularly performs outreach to patients after receiving care to encourage feedback via a third-party vendor. Patients may further opt in to participate in occasional additional opinion surveys on various topics related to UC San Diego Health. In March 2025, the UC San Diego Health Sustainability and Patient Experience teams collaborated to develop and conduct a brief survey on the topic of environmental sustainability in healthcare.

The survey consisted of four questions assessing patients’ attitudes towards environmental health and the importance of sustainability-related practices within healthcare. The questions asked participants to rate the importance of a healthy environment to their personal health, the value they place on environmentally friendly practices within healthcare settings, and their preference for receiving care from an organization committed to environmental health. Responses were collected using Likert scales ranging from *Extremely important* to *Not at all important* or from *Strongly agree* to *Strongly disagree*, depending on the question. All survey questions are provided in the Supplement.

The survey was available in English or Spanish and sent via email. Participants had two weeks to complete the survey, with a reminder message sent halfway through to encourage participation. After those two weeks, the survey was closed, and the results were tabulated. Aggregate responses were made available to UC San Diego Health Patient Experience.

This voluntary survey was administered as part of standard operations to obtain generalized, aggregated patient feedback. It did not constitute research involving human subjects as defined under 45 CFR 46 or 21 CFR 56. Publication of the survey results was reviewed by the UC San Diego Aligning and Coordinating Quality Improvement, Research, and Evaluation (ACQUIRE) Committee, and was deemed to not require Institutional Review Board (IRB) review or approval.

Aggregate demographic information of survey participants – such as age range, gender, and race categories – was available within the survey results. More personal or individual details (such as age, income, zip code, etc.) were not available in aggregate. No individual survey participant information was available.

## 3 Results

The environmental stewardship in healthcare survey was sent to 39,019 patients who had previously volunteered to receive surveys for the health system. Of these, 5,389 (14.5 %) responded. Respondents were 57 % female, and 71 % age 65 and above, with decreasing participation among younger participants. Survey participants were 75 % white, 12 % “other”, 6 % Asian, 3 % Black, 3 % unknown, and 1 % Native American. Information regarding Hispanic/non-Hispanic was not available (Table 1). Most respondents were from Southern California, with some representation from Arizona, Nevada, and other parts of California.

**Table 1:** Survey Respondent Demographics.

**GENDER**	**N (%)**
Female	3,060 (57 %)
Male	2,326 (43 %)
Unknown	3 (<0.1 %)
**Race/Ethnicity**	
White	4,063 (75 %)
Black	162 (3 %)
Asian	323 (6 %)
Native American	54 (1 %)
Other	647 (12 %)
Unknown	140 (3 %)
**Age Range (years)**	
65+	71 %
55–64	16 %
45–54	7 %
35–44	4 %
26–34	2 %
≤25	0 %
**Total**	5389

**Fig. 1:**
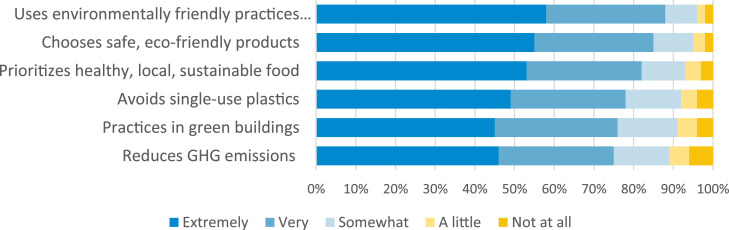
Survey Participants Responses to the Importance of Their Healthcare Facility Engaging in These Sustainable Practices (*N* = 5,389).

The vast majority (94 %) of respondents considered a healthier environment to be extremely or very important to their personal health. Most respondents (88 %) considered it extremely or very important that their healthcare facility uses environmentally friendly practices throughout the facility (Fig. 1). A similar significant majority (85 %) considered it extremely or very important that their healthcare facility chose products that are better for the environment and human health. While 97 % of patients agreed it was important for a healthcare system to provide local, healthy, sustainable food, over 80 % felt it was extremely or very important to do so (Fig. 1).

**Fig. 2:**
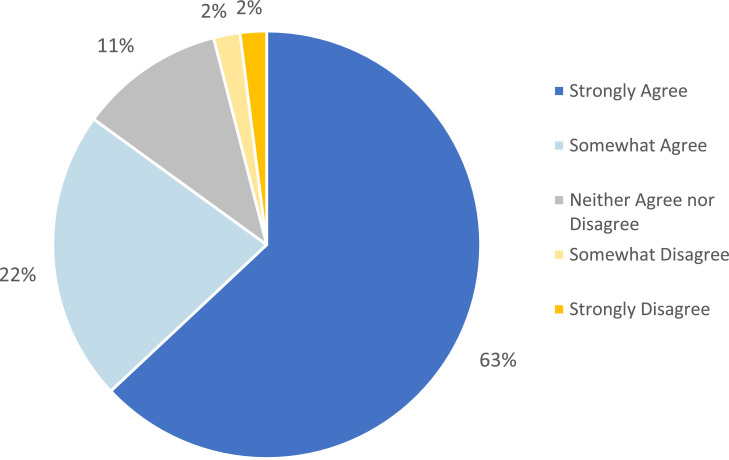
Prefer to Receive Healthcare from an Organization Committed to Environmental Health and Sustainability (*N* = 5389).

Survey participants overwhelmingly responded that it was important that their healthcare organization avoid disposable or single-use plastic items when safe reusable alternatives are available, prioritize renewable energy, natural lighting, and green spaces, and work to reduce or eliminate greenhouse gas emissions (Fig. 1). Finally, 85 % of respondents agreed with the statement that they preferred to receive healthcare from an organization that is committed to environmental health and sustainability (63 % strongly agreed) (Fig. 2).

Despite not eliciting comments, several survey respondents wrote to the UC San Diego Health Sustainability email address with recommendations on areas where they saw opportunities to implement more environmentally conscientious practices, such as reducing packaging materials associated with pharmacy deliveries, reducing paper use in clinic operations, and decreasing chemicals of concern in building operations, among others.

## 4 Discussion

Little has been quantified about how patients view the environment as part of their health, and similarly scant efforts have been made to understand patient perceptions of environmental sustainability in healthcare [13,14]. Patients participating in this survey expressed a strong link between their sense of health and environmental health, and strong support for a health organization’s efforts to curb environmental harm. The U.S. healthcare system contributes 8.5 % of the nation’s greenhouse gas emissions, produces approximately 5.9 million tons of waste each year, and accounts for 8.2 % of national plastic consumption – approximately $30.4 billion worth as of 2022, with usage expected to rise [[15], [16], [17]]. Much of this waste is visible to patients who also are keenly aware of the high cost of healthcare in the United States. This survey provided a platform for patients to share their perceptions of health and the environment, and revealed the value added when a healthcare facility takes steps to reduce their environmental impact. Integrating environmental sustainability into healthcare can promote loyalty among patients, employees, and the broader health system. This survey allowed patients to voice their preferences for environmentally responsible healthcare.

A limitation of this survey is that it was voluntary, so it may have bias towards those who are already interested in environmental stewardship. However, the opposite could also be true, and those with opposing views could have been more motivated to respond. The response rate of 14.5 % was an average response rate compared to other “Community Insights” surveys performed by UC San Diego Health’s Patient Experience group. Another limitation of this survey is that it only reached patients who have regular access to a phone or computer, excluding patients without digital access to care. It was also conducted in English and Spanish, making it less accessible to patients who speak other languages.

While considering the impact of this survey, we recognize that the majority of patients who participated were from California, a state that has been proactively advocating for environmental stewardship. Nonetheless, the strong response supports the value of prioritizing environmental sustainability in healthcare. While this may be perceived as a limitation of the applicability of these findings to geographically diverse locations, we encourage other health centers across the country to give patients the opportunity to communicate on the topic to better understand how environmental sustainability in healthcare is valued by patients.

## 5 Conclusions

Environmental stewardship promotes health for patients both inside and outside of the healthcare setting. Advancing healthcare environmental stewardship requires supportive leadership and dedicated staff to drive meaningful change. These patient survey results should bolster clinicians, healthcare administrators, policymakers, and associated industry partners (such as the pharmaceutical industry and medical device manufacturers) to fully embrace efforts to green operations and promote planetary health.

Patients voiced an unquestionable preference for healthcare providers who align medical care with environmental responsibility. Embracing sustainable practices as a strategic imperative can enhance patient loyalty and support overall health. This strong messaging can and should support healthcare organizations to create environmentally sound operations. Healthcare engagement in sustainability not only supports environmental and public health goals, but can also reflect patient values and is an integral component of patient-centered care.

## CRediT authorship contribution statement

**Shira R. Abeles:** Writing – review & editing, Writing – original draft, Supervision, Project administration, Methodology, Investigation, Formal analysis. **Jennifer Woods:** Writing – review & editing, Visualization, Project administration, Methodology, Formal analysis. **Nicole Poletto:** Writing – review & editing, Visualization, Project administration, Methodology, Formal analysis. **Lesley Wilson:** Writing – review & editing, Project administration, Investigation, Conceptualization. **Monica Nakielski:** Writing – review & editing, Methodology, Conceptualization, Project administration, Investigation, Formal analysis.

## Declaration of competing interest

The authors declare that they have no known competing financial interests or personal relationships that could have appeared to influence the work reported in this paper.

## Appendix Supplementary materials

Image, application 1
