# Supplementary material for: Patients weigh in: The value of healthcare environmental stewardship to patient experience
Source: J Clim Chang Health. 2026 Mar 27;28:100647. doi: 10.1016/j.joclim.2026.100647 (PMC13054402; doi:10.1016/j.joclim.2026.100647)
Supplement: Supplementary file 1 [file mmc1.docx]

**Patient Experience Core Questions: Environmental Stewardship in Healthcare**

1. How important is a healthier environment (meaning clean air, clean water, etc.) to you and your personal health?
   - Extremely important
   - Very important
   - Somewhat important
   - A little important
   - Not at all important
2. How important is it to you that your healthcare facility uses environmentally friendly practices throughout the facilities? Examples of environmentally friendly practices include using healthier materials, reducing waste, and providing healthy and sustainable food in healthcare settings.
   - Extremely important
   - Very important
   - Somewhat important
   - A little important
   - Not at all important
3. How important is it to you that your healthcare facility does the following?
   - Avoids disposable items or single-use plastics when safe reusable alternatives are available
   - Chooses products that are better for the environment and human health
   - Prioritizes providing food that is healthy, local, and sustainable (e.g., meat raised without antibiotics, locally and sustainably grown produce, plant-forward menus, sustainably packaged food)
   - Practices in buildings that prioritize natural lighting, green spaces, and operate off of renewable energy
   - Reduces or eliminates greenhouse gas emissions in healthcare operations and services
     - Extremely important
     - Very important
     - Somewhat important
     - A little important
     - Not at all important
4. How much do you agree or disagree with the following statement?

I prefer to receive healthcare from an organization that is committed to environmental health and sustainability.

- Strongly agree
- Somewhat agree
- Neither agree nor disagree
- Somewhat disagree
- Strongly disagree
